# Stimuli-Responsive Nanocomposite Hydrogels for Oral Diseases

**DOI:** 10.3390/gels10070478

**Published:** 2024-07-18

**Authors:** Raffaele Conte, Anna Valentino, Silvia Romano, Sabrina Margarucci, Orsolina Petillo, Anna Calarco

**Affiliations:** 1 Research Institute on Terrestrial Ecosystems (IRET), National Research Council (CNR), Via Pietro Castellino 111, 80131 Naples, Italy; anna.valentino@cnr.it (A.V.); silviaromano_t@hotmail.it (S.R.); sabrina.margarucci@cnr.it (S.M.); orsolina.petillo@cnr.it (O.P.); anna.calarco@cnr.it (A.C.); 2 National Biodiversity Future Center (NBFC), 90133 Palermo, Italy; 3 Faculty of Medicine and Surgery, Saint Camillus International University of Health Sciences, Via di Sant’Alessandro 8, 00131 Rome, Italy

**Keywords:** nanocomposite hydrogels, drug delivery, oral cancer, periodontitis, endodontic infections

## Abstract

Oral diseases encompassing conditions such as oral cancer, periodontitis, and endodontic infections pose significant challenges due to the oral cavity’s susceptibility to pathogenic bacteria and infectious agents. Saliva, a key component of the oral environment, can compromise drug efficacy during oral disease treatment by diluting drug formulations and reducing drug-site interactions. Thus, it is imperative to develop effective drug delivery methods. Stimuli-responsive nanocomposite hydrogels offer a promising solution by adapting to changes in environmental conditions during disease states, thereby enabling targeted drug delivery. These smart drug delivery systems have the potential to enhance drug efficacy, minimize adverse reactions, reduce administration frequency, and improve patient compliance, thus facilitating a faster recovery. This review explores various types of stimuli-responsive nanocomposite hydrogels tailored for smart drug delivery, with a specific focus on their applications in managing oral diseases.

## 1. Introduction

The oral cavity presents a unique environmental niche, with its front part exposed to the external environment and its rear part connected to the digestive and respiratory tracts [1]. This complex environment is conducive to the growth of various bacteria, including *Porphyromonas gingivalis* (*P. gingivalis*), *Fusobacterium nucleatum*, and *Streptococcus mutans* (*S. mutans*) [2,3]. These bacteria have the potential to disturb the equilibrium between host immunity and microbial proliferation, thereby causing various conditions including periodontitis and even oral cancer [4,5]. In particular, oral dysbiosis, which disrupts the balance between host immunity and bacterial growth, is the main cause of periodontitis [6,7]. Differently, genetic alterations in host epithelial cells, caused by bacterial metabolites, cause cell proliferation and the inhibition of apoptosis and migration, which can lead to oral cancer [8]. Treating oral disorders with pharmaceutical agents requires consideration of the unique environment and the microbiota of the oral cavity. Although systemic drug delivery methods, such as intravenous and oral administration, are commonly used in clinical settings, they are not advised for oral diseases because of low drug cumulation at the target site and toxicity [9]. Systemic drug administration can damage nontarget tissues and organs, leading to drug resistance and dysbiosis. In contrast, targeted delivery in the oral cavity can reduce systemic toxicity. However, the practical application of local delivery is challenging because factors such as first-pass metabolism, salivary flow, drug dilution, and digestive enzymes can reduce bioavailability and efficacy [10]. Additionally, the limited water solubility and poor permeability of certain drugs may restrict their absorption efficiency following direct local administration [11]. Therefore, the development of strategies for precise, targeted, sustained, and effective drug delivery is crucial. Emerging material-based drug delivery systems offer promising solutions because they can regulate drug release, achieving precise localized delivery at the target site, thereby enhancing pharmacological activity and improving bioavailability [12]. These systems enhance efficacy and reduce adverse effects by incorporating drugs into a suitable carrier through dissolution, embedding, wrapping, or attachment methods. Innovative biomaterial carriers (for example, tridimensional scaffolds, thin films, hydrogel, nanoparticles, and nano-fibers) are effective drug delivery systems for the treatment of oral diseases [13]. Hydrogels, in particular, have garnered significant attention against oral disorders owing to their excellent biocompatibility, drug delivery ability, and reduced cost. Hydrogels also exhibit exceptional bio-adhesivity, which allows them to adhere to oral tissues and enables sustained drug release [14]. These properties make hydrogels promising platforms for oral drug delivery. Hydrogels are structured gels that contain sulfate (–SO_3_H), amino (–NH_2_) and carboxyl (–COOH) groups, composed of hydrophilic polymers such as polysaccharides or peptides. This composition allows the delivery of both hydrophilic and hydrophobic drugs [15]. The physicochemical properties of hydrogels are determined by their constituents that confer diverse functions. The addition of nanosized structures such as nanocomposite hydrogels can lead to stimuli responsiveness. Stimuli-responsive or environmentally sensitive nanocomposite hydrogels are able to adapt their characteristics in relation to physicochemical stimuli [16]. Traditional hydrogels control drug release through changes in gel structure (swelling, dissolution, or degradation), whereas stimuli-responsive nanocomposite hydrogels can be designed to respond to specific features of the target site, allowing for manageable drug release [16]. Such “smart” hydrogels have a dual control on the area and time of drug delivery, offering significant advantages for the treatment of oral pathologies [17]. For example, thermosensitive nanocomposite hydrogels can change their gelation and solution conditions in response to temperature stimuli, enabling an in vitro non-destructive drug loading, with rapid in vivo gel formation and the ability to create a drug reservoir [18,19]. Moreover, stimuli-responsive nanocomposite hydrogels can be synthesized to specifically match the characteristics of target lesions, such as the acidic microenvironment of oral tumors, to reduce the off-target effects on normal tissues. In such cases, pH-sensitive hydrogels are preferred for precise drug release control to prevent systemic side reactions [17]. Many studies identified stimuli-responsive nanocomposite hydrogels as promising drug delivery systems for oral disease treatment. The aim of this review is to highlight emerging trends in this field that have not been thoroughly reviewed previously. This encompasses new stimuli-responsive mechanisms and innovative drug-loading strategies.

## 2. Classification of Stimuli-Responsive Nanocomposite Hydrogels

Stimuli-responsive nanocomposite hydrogels play a crucial role in addressing reduced activities in living tissues and are emerging as pivotal solutions to promote overall well-being, particularly in the context of oral diseases [20,21]. Through intelligent design, these platforms can effectively respond to stimuli such as temperature, pH, redox, enzymatic activity, and light exposure [22]. Within the existing repertoire of biomaterials, hydrogels and nanoparticles are notable because of their inherent design flexibility and the ease with which they can be tailored to incorporate stimuli-responsive features. These features make them highly versatile for a wide range of applications, including stem cell therapy, drug delivery, and biosensing [23,24]. Hydrogels are exceptional scaffolds for fostering tissue formation, demonstrating the ability to replicate crucial features of the native extracellular matrix (ECM) [25]. Conversely, nanoparticles are well-suited to function as efficient reservoirs for bioactive molecules, accommodating both hydrophilic and hydrophobic compounds with efficacy [26,27]. In particular, organic [28,29,30], inorganic [31], and silicate nanoparticles [32] are utilized in the development of materials with enhanced mechanical strength, whereas metal ions and rare earth elements contribute to the creation of nanoparticles with distinctive magnetic, optical, or catalytic features [33]. The fusion of these two classes results in hybrid platforms, referred to as nanocomposite hydrogels. These innovative structures seamlessly incorporate the beneficial characteristics of both systems, effectively mitigating the individual limitations associated with each system [34]. The integration of nanoparticle elements into hydrogel matrices not only broadens their spectrum of attainable mechanical properties, but also improves local nanoparticle retention [35]. Additionally, these hybrid materials offer valuable functionalities, such as tissue adhesivity, tailored biodegradation, and responsiveness to electrical, thermal, and light stimuli. The incorporation of multiple stimuli-sensitive elements into nanocomposite hydrogels further expands their properties, allowing the creation of highly sophisticated bifunctional materials capable of responding to various inputs and generating several outputs in diverse contexts [35]. In the realm of stimuli-responsive delivery, intrinsic stimuli that naturally exist within the human body have garnered extensive exploration [36]. These include the pH, redox potential, and enzyme activity. Under physiological conditions, these stimuli are ubiquitous, and some become highly dysregulated in pathological states, offering opportunities for smart platforms to autonomously execute effective therapeutic interventions by distinguishing between healthy and afflicted sites. The adaptability of stimuli-responsive nanocomposite hydrogels is further highlighted by their ability to be tuned for threshold sensitivity, enabling responses in both physiological and pathological states [36]. Conversely, nanocomposite hydrogels are tailored to react to extrinsic stimuli that are absent in tissues, unless externally applied. Such cues encompass temperature and light irradiation [37]. This engineering approach allows the creation of platforms that remain unchanged in biological environments but react when an external cue is applied [37].

### 2.1. pH Responsive

Modification of pH is a common characteristic observed in various pathological conditions including oral diseases, tumors, and inflammation [38]. Platforms that respond to changes in pH demonstrate the ability to dynamically adjust their structural properties in response to the acidic pH prevalent in these pathological states. This adaptability facilitates the targeted delivery of bioactive molecules to specific sites affected by the disease, thereby enhancing the precision of therapeutic interventions [39]. In addition to pathophysiological dysregulation, pH levels undergo characteristic alterations at specific anatomical sites, such as mouth saliva [40]. Two primary approaches are commonly employed for the design of pH-responsive nanocomposite hydrogel materials. The first involves incorporating pH-ionizable moieties that change their charges based on the pH of the surrounding status, as determined by their characteristic pKa. The second approach involves the incorporation of pH-cleavable covalent bonds, typically acid-labile bonds. The specific pKa of ionizable groups is influenced by neighboring groups in the drug/polymer backbones, like the conformation of surrounding components in the 3D mesh [41].

### 2.2. Redox Responsive

The redox potential, representing the reduction–oxidation (redox) state, is a critical biological parameter susceptible to alterations in various conditions and is often found to be disturbed in specific diseases, such as cancer and inflammation, or in hypoxic conditions [42,43]. Similar to the pH gradients within the intracellular area, redox potential is settled through elevated glutathione concentrations in the cytosol and endocytic organelles, providing a unique avenue for triggering intracellular drug delivery. Redox-responsive nanocomposite hydrogels typically use reduction-cleavable linkers (e.g., disulfide, diselenide, or thiol-maleimide), reactive oxygen species-sensitive moieties (e.g., diselenide, phenylboronic ester, thioketal, or thioether), or metallic nanoparticles [44].

### 2.3. Enzyme Responsive

Enzymes play a crucial role throughout the human body and participate in the essential biological processes necessary for the survival of living organisms. Their ubiquitous presence, coupled with their specific involvement in various tissues and potential overexpression in certain disease states, provides numerous opportunities for the design of controlled drug delivery platforms. Enzyme-responsive systems offer advantages in terms of specificity and efficiency compared to conventional stimuli, such as pH, temperature, or light, making them ideal candidates for precise and sophisticated drug delivery applications [45]. Enzyme-responsive nanocomposite hydrogels can be fabricated using sensitive natural polymers (such as fibrin, collagen, gelatin, and hyaluronic acid) or by modifying inert biomaterials with enzyme-sensitive linkers [46].

### 2.4. Thermo Responsive

Temperature is a widely explored stimulus in the design of stimuli-responsive platforms, owing to its mild and convenient application. Thermoresponsive polymeric materials such as methyl cellulose derivatives, poly(N-isopropylacrylamide), and poloxamers undergo phase transitions in response to temperature changes [47]. Genetic engineering tools can also create biomimetic synthetic proteins with tailorable temperature-responsiveness [48]. A human body temperature of approximately 37 °C allows the design of platforms to sense intra-body heat, resulting in self-dissolving platforms or in situ hydrogel formation [49]. Thermoresponsive nanocomposite hydrogels offer emerging applications in drug delivery, shape memory actuation, sacrificial materials for bio-printing, and biosensing/bioimaging using quantum dot nanoparticles as cross-linkers [50].

### 2.5. Light Responsive

The utilization of light as an external stimulus has become increasingly attractive in the design of advanced biomedical platforms that offer enhanced spatiotemporal control over biomaterial behavior [51,52]. Light-responsive systems not only provide the capability for remote-controlled therapeutic delivery, but also enable the sequential degradation of implantable devices in a manner that is both safe and non-invasive. There are two predominant approaches in the design of light-responsive systems: those based on photodegradable hydrogel networks that encase bioactive cues or nanomaterials, and those relying on photoreversible interactions [53,54]. Photodegradable hydrogel networks, employing moieties such as nitrobenzyl esters, can encapsulate bioactive cues or nanomaterials that are prompted to be released on-demand under light stimulation. On the other hand, hydrogel networks relying on photoreversible interactions, such as the host–guest system azobenzene-cyclodextrin, the dimerization/cycloaddition of coumarin/anthracene, or engineered proteins, allow for the softening/stiffening switch or the cyclic assembly/disassembly change. The potential and design flexibility of these systems can be significantly enhanced by incorporating nanoparticles with antennae-like capabilities into nanocomposite hydrogel formulations [55].

**Figure 1:**
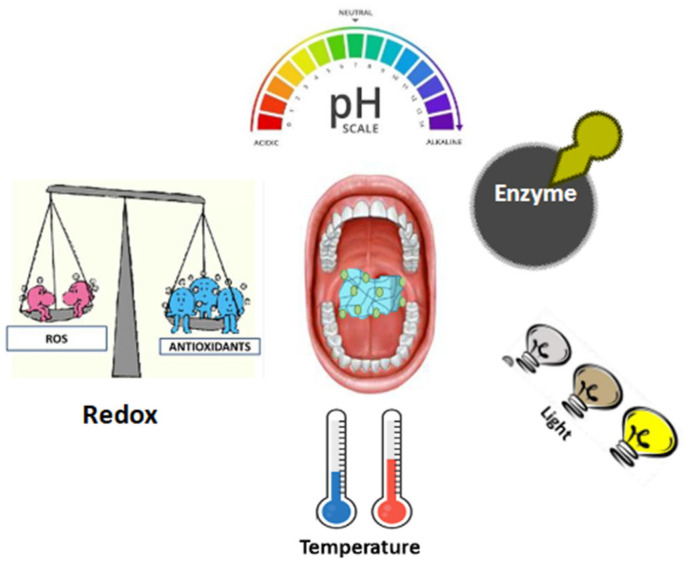
Stimuli-responsive nanocomposite hydrogels An image with 600 dpi and 1400 pixel was added.

Figure 1 provides an overview of the stimuli applied to the materials designed for oral cavity applications.

## 3. Stimuli-Responsive Nanocomposite Hydrogels for Periodontitis Treatment

**Figure 2:**
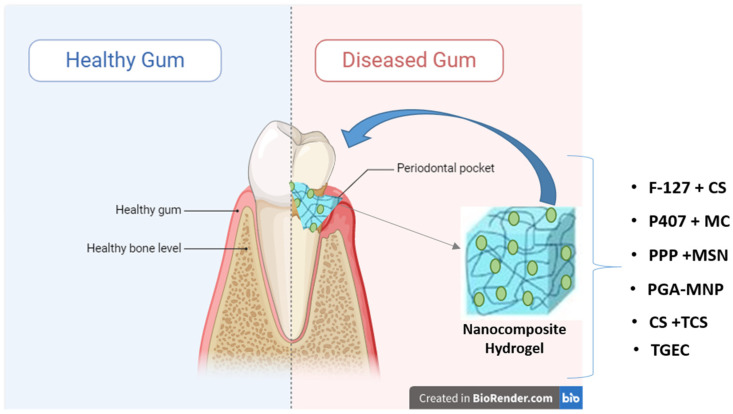
Stimuli-responsive nanocomposite hydrogels for periodontitis treatment.

Periodontitis is a widespread chronic inflammatory condition characterized by progressive destruction of tooth-supporting tissues, including the gums, periodontal ligaments, and alveolar bone. It typically results from the interaction of pathogenic bacteria with the host immune and inflammatory responses, compounded by environmental factors such as poor oral hygiene and smoking. Genetic factors also play a role, with certain individuals being predisposed to developing periodontitis [56]. Conventional treatment approaches for periodontitis often involve mechanical debridement of bacterial biofilms and adjunctive use of antibiotics to combat microbial infections. However, the systemic administration of antibiotics presents challenges, including the risk of antibiotic resistance and disruption of the body’s microbiota [57,58]. Localized drug delivery systems, particularly those based on stimuli-responsive nanocomposite hydrogels, are promising alternatives. These hydrogels can be engineered to respond to specific environmental cues, such as changes in temperature, pH, or enzymatic activity, enabling the controlled release of therapeutic agents directly at the site of infection (Figure 2).

For example, Chen et al. [59] produced a thermoresponsive, osteotropic, injectable hydrogel based on pyrophosphorolated pluronic F127 for the local delivery of Simvastatin (SIM). The erosion of this device produces nanosystems delivering SIM with effective anti-inflammatory and bone anabolic properties, which have shown promising benefits in mitigating periodontal bone loss in vitro and in a rat model [59]. Valentino et al. introduced a localized drug delivery system combining Hyt-loaded chitosan nanoparticles (Hyt-NPs) with in situ-forming hydrogels to leverage the advantages of both. This thermosensitive formulation, utilizing Pluronic F-127 (F-127), hyaluronic acid (HA), and Hyt-NPs (referred to as Hyt@tgel), demonstrated a distinctive capability to be injected in a minimally invasive manner into a targeted area as a liquid solution at room temperature, transforming into a gel upon reaching body temperature [60]. Pham et al. [61] employed Poloxamer 407, a triblock copolymer consisting of ethylene oxide and propylene oxide (PEO-PPO-PEO), for local injection into the periodontal pocket to sustain the effective release of SIM. However, Poloxamer 407 exhibits restricted mechanical durability and instability under physiological conditions. To improve the properties of poloxamer 407, a composite hydrogel incorporating nanostructures of methylcellulose (MC) and silk fibroin (SF) was developed for in situ temperature-sensitive applications. This study showed that MC creates hydrogen links with Poloxamer 407, which enhances the network structure of the gel and increases its viscosity. Additionally, SF contributed to a rigid β-sheet structure, which further strengthened the poloxamer 407 matrix. This enhanced the encapsulated metronidazole (MTZ) within its hydrophobic core. At the body temperature of 37 °C, the hydrogel quickly transitioned into a gel state in the periodontal pocket. The initial concentration of released MTZ was higher than the minimum inhibitory concentration (MIC) required to combat *P. gingivalis*, and the MTZ release was sustained for up to ten days, reducing the necessity for frequent dosing [61]. Beyond antibiotics, such nanocomposite hydrogels can also be used to deliver baicalin and clove oil to form an in situ drug reservoir. Baicalin and clove oil help in the repair of periodontal tissue, reduce osteoclast aggregation, and promote collagen regeneration [62]. Periodontitis is often associated with diabetes mellitus (DM). The combination of mesoporous silica nanoparticle (MSN)-incorporated PDLLA-PEG-PDLLA (PPP) temperature-sensitive hydrogels offers dual benefits. These nanocomposite hydrogels not only enhance bone regeneration but also aid in diabetes management. The MSN-incorporated PPP hydrogels were developed by integrating the thermosensitive copolymer poly(D,L-lactide)-block-poly (ethylene glycol)-block-poly(D,L-lactide) (PDLLA-PEG-PDLLA) with MSNs loaded with metformin and stromal cell-derived factor-1 (SDF-1), which mimics the natural bone-healing process in diabetic periodontal regeneration. These devices have a phase transition temperature of 35 °C, allowing them to transform into a gel state upon injection into the periodontal pocket. The release of SDF-1 from hydrogels stimulated the recruitment and accumulation of bone marrow stem cells (rBMSCs) in injured periodontal areas. Additionally, the slow degradation of the hydrogels exposed MSNs. In a high-blood-sugar environment, metformin-loaded MSNs reduce reactive oxygen species (ROS) and enhance bone regeneration capabilities [63]. Chitosan-based thermosensitive injectable self-assembled hydrogels loaded with resveratrol and nanosized granulocyte–macrophage colony-stimulating factor (GM-CSF) have been developed to induce a tolerogenic phenotype in dendritic cells and alleviate periodontal inflammation [64]. Hydrogels based on this material showed promise in treating periodontitis by leveraging diverse stimuli-responsive mechanisms to deliver various drugs, resulting in varying degrees of therapeutic efficacy [64]. For example, the ability of chitosan to respond to pH variations was exploited by Aminu et al. [65], who developed a dual action, namely anti-inflammatory and antimicrobial, nanocomposite hydrogel for the treatment of periodontitis using triclosan (TCS) and flurbiprofen (FLB). Triclosan, an antimicrobial drug, was prepared as nanoparticles using poly-ε-caprolactone (PCL), while flurbiprofen, an anti-inflammatory drug, was directly loaded into a chitosan (CS) hydrogel [65]. The swelling degree of the chitosan hydrogels was influenced by pH, with an inverse relationship between the swelling ratio and pH value. Additionally, the percentage of erosion of pH-sensitive chitosan hydrogels containing triclosan-loaded nanoparticles exceeded 80% at pH 5.64. This demonstrates their significant potential for stable TCS release in periodontal pockets [65]. Chitosan pH-sensitive hydrogels have also been produced to deliver N-phenacylthiazolium bromide (PTB) with a higher release rate of PTB at pH 5.5 [66]. pH-sensitive hydrogels containing methacryloil-group-modified (MPGA)/methacrylated-poly γ-glutamic acid nanoparticles (PGA-MNPs) were synthesized using blue light photopolymerization. The MPGA/PGA-MNP hydrogels were capable of loading metronidazole and chlorhexidine (CHX). The release of CHX from these hydrogels was significantly dependent on pH without affecting its activity. CHX is crucial for reducing the activity of MMPs in *P. gingivalis*, demonstrating strong antibacterial activity [67]. Qi et al. transformed the Turkish Galls effective constituent (TGEC, T) into nanoparticles (T-NPs) through oxidative self-polymerization. These pH-sensitive T-NPs were then loaded in a thermosensitive hydrogel. Bacteriostatic testing revealed that Turkish Galls nanoparticles exhibited higher antibacterial activity against oral pathogens than TGEC alone. Specifically, the minimum inhibitory concentration (MIC) of T-NPs against *P. gingivalis* and *A. viscosus* was reduced by 50% and 25%, respectively, compared to that of TGEC [68]. Table 1 reviews the nanocomposite hydrogels used for periodontitis treatment.

## 4. Stimuli-Responsive Nanocomposite Hydrogels for Oral Tumor Therapy

**Figure 3:**
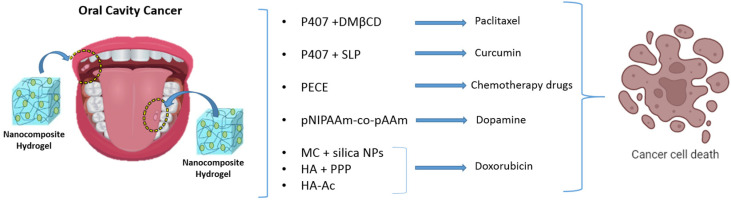
Stimuli-responsive nanocomposite hydrogels for cancer treatment.

In 2012, there were an estimated 300,000 cases of oral tumors worldwide, with approximately 145,000 deaths resulting in a mortality rate of approximately 48.3% [69]. Each year, 50,000 new cases of oral cancer are globally reported [69]. Even though there have been improvements in the diagnosis and treatment of oral tumors, the 5-year survival rate remains around 50% [69]. Oral squamous cell carcinoma (OSCC) is the predominant type, accounting for approximately 90% of all oral tumor cases [70]. The treatment of these tumors is significantly hindered by the severe side effects and drug resistance associated with chemotherapeutic drugs. As a result, there is growing interest in the use of stimuli-responsive nanocomposite hydrogel-based drug delivery systems for treating oral tumors. The temperature in the region of the oral tumor was consistently higher than that of the surrounding mucous membrane. This makes temperature-sensitive nanocomposite hydrogels highly promising for oral cancer treatment (Figure 3).

Paclitaxel, a common anticancer drug used to treat oral cancer, faces significant challenges owing to its poor water solubility and severe side effects [71]. However, combining paclitaxel with nano (2,6-di-O-methyl)-β-cyclodextrin (DMβCD), polyoxyethylene, and poloxamer 407 can localize the drug to the mucous membrane and extend its interaction with the tumor [72]. In a further study, researchers developed poloxamer 407 temperature-sensitive hydrogels containing curcumin-loaded lipid-core nanocapsules (C-SPNs) coated with chitosan [73]. These hydrogels can transition to a gel state below 37 °C, maintaining gelation at oral temperatures. C-SPNs improved the adhesion to the oral mucosa and significantly reduced the activity of epidermoid cancer cells [73]. Although poloxamer 407 temperature-sensitive hydrogels are effective for stable drug release from oral tumors, their duration is limited. Poly(ethylene glycol)-poly(ε-caprolactone)-poly(ethylene glycol) (PEG-PCL-PEG, PECE) hydrogels were developed with the ability to remain effective in the oral cavity for more than 14 days when administered via subcutaneous injection, which is significantly longer than the duration of poloxamer 407 hydrogels. Additionally, PECE hydrogels exhibit excellent biocompatibility, biodegradability, and temperature sensitivity [74]. Temperature-sensitive hydrogels combined with pH-sensitive hydrogels can achieve synergistic therapy via photothermal therapy (PTT) and multidrug chemotherapy. This synergistic anti-cancer system was created by integrating pNIPAAm-co-pAAm temperature-sensitive hydrogels with dopamine nanoparticles loaded with bortezomib and doxorubicin [75]. Dopamine nanoparticles act as potent photothermal agents that convert light energy into heat. This heat not only destroys cancer cells but also induces the sol–gel transformation of pNIPAAm-co-pAAm hydrogels, leading to the gradual release of doxorubicin [76]. Additionally, the acidic tumor microenvironment triggered the separation of catechol groups in the dopamine nanoparticles from the boronic acid functionality of bortezomib, thereby enhancing the efficacy of the drug. Su et al., in fact, produced a PEG based system containing these vehicles [77]. Photodynamic therapy (PDT) and photothermal therapy (PTT) are innovative methods for treating oral cancers. Exogenous light stimuli can be used to design environmentally sensitive hydrogels that specifically target oral cancers. Traditional phototherapy typically involves intravenous injection of a photothermal agent, which often has low biocompatibility and does not fully accumulate in the tumor region [78]. Consequently, there is a significant need for light-sensitive nanocomposite hydrogels that can precisely localize to the tumor site. Yongzhi Wu developed an injectable hybrid system that utilizes diselenide-bridged doxorubicin-loaded mesoporous silica nanoparticles (MSNs), combined with IR820-packaged methylcellulose (MC) hydrogels, as a photothermal agent with NIR light-responsive abilities [79]. Upon exposure to NIR light, this hybrid system generates substantial thermal energy to eradicate oral cancer cells. Additionally, NIR light induces the generation of excess reactive oxygen species (ROS), which then breaks down the redox-sensitive diselenide bonds, facilitating the release of doxorubicin [79]. In addition to temperature- and light-sensitive hydrogels, metalloproteinase (MMP)-sensitive hydrogels can be utilized in the treatment of oral cancer. MMPs exhibit specificity not only in periodontal pockets but also in oral tumors. Studies have shown that the levels of latent, active, and total forms, as well as the activation ratio of MMP-2 and MMP-9, are significantly higher in oral tumors than in surrounding normal tissues [80]. MMP-sensitive hydrogels were created by cross-linking an MMP-responsive peptide (with the structure GCRDGPQGIWGQDRCG) with hyaluronic acid hydrogels loaded with doxorubicin encapsulated within biodegradable poly esther-based (PDLLA-PEG-PDLLA) nanomicelles [81]. In the metalloproteinase-rich microenvironment of oral cancer, these hydrogels exhibit a high drug release and degradation. Furthermore, MMP-sensitive hydrogels have demonstrated elevated toxicity against oral cancer cells and effectively inhibit tumor expansion [81]. Yuan-huan Wang successfully developed nano doxorubicin-indocyanine green (ICG) matrix metalloproteinase-responsive hydrogels by combining ICG with hyaluronic acid-acrylate (HA-Ac) hydrogels [82]. In a mouse model of squamous cell carcinoma, these MMP-responsive hydrogels fully degraded within three weeks, whereas in normal tissue, 40% of the hydrogels remained intact. Additionally, upon laser exposure, the temperature of this system rose to 65.6 °C, producing a substantial amount of reactive oxygen species (ROS), which significantly contributed to the ablation of head and neck tumors [82]. Table 2 outlines the nanocomposite hydrogels used for oral tumor therapy.

## 5. Stimuli-Responsive Nanocomposite Hydrogels for the Treatment of Endodontic Infections

**Figure 4:**
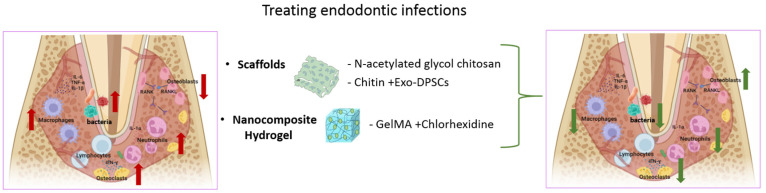
Stimuli-responsive nanocomposite hydrogels for endodontic infections treatment.

The dental pulp, located within the pulp chamber and surrounded by dentin, possesses the unique ability to generate dentin and maintain blood circulation, thus facilitating dentin regeneration [83]. Typically, the pulp chamber is sealed to prevent bacterial invasion. However, when dental hard tissues are compromised by factors such as caries, trauma, or cracks, bacteria can penetrate the pulp chamber, leading to pulp infection, endodontitis, or necrosis [84]. Root canal therapy is a commonly employed clinical method to treat infected dental pulp by removing the diseased tissue and controlling bacterial infections. Despite its effectiveness, symptoms may worsen in cases where the pulp-dentin complex is compromised, necessitating tooth extraction [85]. The success of root canal therapy depends on factors such as the extent of residual pulp tissue, tooth integrity, and bacterial clearance (Figure 4).

To overcome the constraints associated with conventional root canal treatment, regenerative endodontic procedures (REPs) and anti-inflammatory therapies were developed. REPs utilize tissue engineering techniques to promote differentiation of the pulp–dentin complex using stem cells, scaffolds, and growth factors [86,87]. Nanocomposite hydrogels play a crucial role in this process by regulating cellular behavior, providing scaffolds for stem cells, releasing growth factors, and reducing inflammation. Owing to the presence of bacteria, the microenvironment of the diseased pulp becomes acidic. Intractable endodontic infections are closely associated with MMP. Thus, developing pH-sensitive nanocomposite hydrogels tailored to the pH level of the diseased pulp and sensitive to MMP action is a novel strategy for treating endodontic infections. Juliana S. Ribeiro pioneered the development of MMP-sensitive GelMA hydrogels, which exhibit efficacy in ablating endodontic infections [88]. GelMA contains MMP degradation sites, which can be enhanced by nanotubes. Their research indicated that nanotube-modified GelMA hydrogels can encapsulate chlorhexidine and act as reservoirs for this antimicrobial agent. Upon exposure to infectious root canals, nanotube-modified GelMA hydrogels degraded in response to MMP stimulation, facilitating the controlled release of chlorhexidine to alleviate inflammation [88]. Chitin-based hydrogels encapsulating dental pulp stem cells (DPSCs)-derived nano exosomes were produced by Wang et al. to enhance the formation of pulp-like tissue within the root canal [89]. Chitin-based hydrogels can also expedite the odontogenic differentiation of DPSCs. Then, by N-acetylating glycol chitosan, glycol chitin-based hydrogels attained temperature sensitivity, undergoing rapid sol-gel transformation at 37 °C that promoted the elevated expression levels of dentin sialophosphoprotein and dentin matrix protein-1, biomarkers indicative of odontogenic differentiation [90]. Table 3 shows the nanocomposite hydrogels used for endodontic infection treatment.

## 6. Limitations Associated with the Utilization of Stimuli-Responsive Nanocomposite Hydrogels

Stimuli-responsive nanocomposite hydrogels hold immense promise for oral disease therapy. However, their application still requires extensive exploration. Currently, most studies are based on cell and animal experiments, with limited translation into clinical trials or market availability. Comprehensive preclinical studies are then essential to understand the long-term biocompatibility and stability of these materials in the oral environment [91]. Additionally, due to continuous exposition to mechanical forces and to diverse microbial communities, drug-loaded devices have limited exposure times, posing a challenge in designing nanocomposite hydrogels suitable for the entire treatment process. The appropriate concentration, drug load, utilization frequency, and cytotoxic effects of stimuli-responsive nanocomposite hydrogels remain unclear and require further confirmation through clinical trials [91]. Despite the general perception of low toxicity, long-term application of these devices may trigger immune-mediated foreign body responses (FBRs), impacting their biocompatibility. Therefore, a nuanced understanding of the used materials is essential to validate their safety and efficacy in humans. These trials should focus not only on the therapeutic outcomes, but also on any potential side effects or complications that may arise from long-term use [92]. In another case, the synthesis and gelation processes are intricate, time-consuming, and costly, hindering the commercial mass production and large-scale application of nanocomposite hydrogels. Then, the scalability of hydrogel production and the reproducibility of their stimuli-responsive behavior must be rigorously tested to ensure consistent performance across different batches [93]. Finally, the regulatory pathways for approval of these advanced materials also require careful navigation, necessitating collaboration between researchers, clinicians, and regulatory bodies to establish standardized protocols for evaluation [94].

## 7. Conclusions and Future Remarks

This review presented a comprehensive overview of stimuli-responsive nanocomposite hydrogels, encompassing their concept, classification and advantages, particularly in the context of oral diseases treatment. Nanocomposite hydrogels are extensively utilized in drug delivery across various medical conditions due to their excellent biocompatibility, ease of preparation, and minimal toxicity. Stimuli-responsive nanocomposite hydrogels offer several advantages, including controlled drug release rates, prevention of salivary interference to prolong drug efficacy, reduced need for frequent drug administration, and improved patient compliance. Given the unique environment of the oral cavity, a diverse array of nanocomposite hydrogels sensitive to oral stimuli have been developed. These devices can respond to various stimuli and can be combined with different substances to create stimuli-responsive materials with superior properties. Stimuli-responsive nanocomposite hydrogels can integrate drug therapy, photodynamic therapy (PDT), photothermal therapy (PTT), magnetic therapy, and other common oral disease treatment methods to expedite recovery. In conclusion, the future of stimuli-responsive nanocomposite hydrogels for oral disease treatment holds immense potential, driven by advancements in material design, multifunctional therapeutic platforms, and personalized medicine. Focused research on novel nanomaterials, such as polymeric and metallic nanoparticles, can enhance hydrogel responsiveness and efficacy. The interdisciplinary partnerships across materials science, dentistry, and pharmacology will lead to personalized, comprehensive treatment solutions, revolutionizing oral disease management and improving patient outcomes.

## Figures and Tables

**Table 1:** Nanocomposite hydrogels used for periodontitis treatment.

Nanocomposite Hydrogel Composition	Action	Main Finding	Reference
Pluronic F-127 hydrogel containing chitosan nanoparticles (Hyt-NPs)	in situ forming hydrogel for localized drug delivery of Hydroxytyrosol	Efficient and sustained Hyt delivery for osteoarthritis treatment.	[60]
Poloxamer 407 combined with Methylcellulose (MC), and Silk Fibroin (SF) nanostructures	Delivery of metronidazole (MTZ) in periodontal pocket	Local delivery of the drug to the oral infection site.	[61]
PDLLA-PEG-PDLLA (PPP) with Metformin-loaded Mesoporous Silica Nanoparticles (MSNs)	Temperature-sensitive devices for diabetic periodontal regeneration	Emulation of the mesenchymal stem cell “recruitment-osteogenesis” cascade for diabetic periodontal bone regeneration.	[63]
Chitosan hydrogel with Triclosan (TCS) Nanoparticles	pH-sensitive hydrogels for anti-inflammatory and antimicrobial treatment of periodontitis	Dual antibacterial and anti-inflammatory effects with an excellent therapeutic outcome	[65]
MPGA/Methacrylated-poly-γ-glutamic Acid Nanoparticles (PGA-MNP)	pH-sensitive hydrogels with metronidazole and chlorhexidine (CHX) for antibacterial activity	Fast photopolymerizable system which can be loaded with the required amount of medicines and can reduce the side effects of the systemic use of drugs	[67]
Turkish Galls Effective Constituent (TGEC) Nanoparticles (T-NPs) in in situ hydrogel	pH-sensitive and thermosensitive in-situ hydrogel for antibacterial activity	Green solution to prepare nanospheres with natural polyphenols	[68]

**Table 2:** Nanocomposite hydrogels used for oral tumor therapy.

Nanocomposite Hydrogel Composition	Action	Main Finding	Reference
(2,6-di-O-methyl)-β-cyclodextrin (DMβCD) in poloxamer 407 hydrogel	Localized paclitaxel delivery to mucous membrane for extended interaction with oral tumors	Improvement of the in vitro release and cytotoxic effect of paclitaxel.	[72]
Curcumin-loaded lipid-core nanocapsules coated with chitosan in poloxamer 407 hydrogel	Temperature-sensitive hydrogels for improved adhesion to oral mucosa and reduced activity of epidermoid oral cancer cells	mucoadhesive system with potential to deliver buccal treatments.	[73]
PEG-PCL-PEG (PECE) hydrogels with nanoparticles	Long-lasting temperature-sensitive hydrogels for oral cavity application, effective for over 14 days	Excellent thermosensitivity and biodegradability	[74]
pNIPAAm-co-pAAm hydrogels with dopamine nanoparticles	Synergistic therapy for oral cancer through photothermal therapy (PTT) and multidrug chemotherapy	Enhanced cellular uptake and subsequently greater reactive oxygen species (ROS) production upon laser irradiation	[75,76]
Diselenide-bridged doxorubicin-loaded mesoporous silica nanoparticles with IR820-packaged methylcellulose hydrogels)	Photothermal agent generating thermal energy and reactive oxygen species (ROS) upon NIR light exposure to target oral cancer cells	The combination of chemotherapy and phototherapy gives a long-lasting synergistic anti-tumor effect	[79]
MMP-responsive peptide cross-linked hyaluronic acid hydrogels with doxorubicin-loaded PDLLA-PEG-PDLLA nano micelles	High rate of drug release and degradation in the MMP-rich microenvironment of oral cancer	Growth inhibition of oral squamous cell carcinoma without any damage to the organs	[81]
Doxorubicin-ICG matrix MMP-responsive nanoparticles in hyaluronic acid-acrylate (HA-Ac) hydrogels	Enhanced ROS production and tumor ablation upon laser exposure	Favorable synergistic antitumor efficacy and acceptable biosafety	[82]

**Table 3:** Nanocomposite hydrogels used for endodontic infection treatment.

Nanocomposite Hydrogel Composition	Action	Main Finding	Reference
MMP-sensitive GelMA hydrogels with nanotubes	Encapsulation and controlled release of chlorhexidine in response to MMP stimulation, alleviating inflammation in endodontic infections	Inhibition of bacterial growth with minimal cell toxicity	[88]
Chitin-based hydrogels encapsulating DPSCs-derived nano exosomes	Enhancement of pulp-like tissue formation within the root canal	Functional device that uses exosomes as biomimetic tools for tissue engineering	[89]
N-acetylated glycol chitosan (glycol chitin-based hydrogels)	Temperature-sensitive gel promoting odontogenic differentiation of Dental Pulp Stem Cells (DPSCs) and elevated expression levels of dentin sialophosphoprotein and dentin matrix protein-1, biomarkers of odontogenesis	This material improves the hDPCs ability to generate clonogenic adherent cell clusters.	[90]
